# Cerebral Circulation Time is Prolonged and Not Correlated with EDSS in Multiple Sclerosis Patients: A Study Using Digital Subtracted Angiography

**DOI:** 10.1371/journal.pone.0116681

**Published:** 2015-02-13

**Authors:** Lucia Monti, Donatella Donati, Elisabetta Menci, Samuele Cioni, Matteo Bellini, Irene Grazzini, Sara Leonini, Paolo Galluzzi, Sauro Severi, Luca Burroni, Alfredo Casasco, Lucia Morbidelli, Emiliano Santarnecchi, Pietro Piu

**Affiliations:** 1 Unit of Neuroimaging and Neurointervention, Department of Neurological and Sensorial Sciences Azienda Ospedaliera Universitaria Senese, Santa Maria alle Scotte General Hospital, Siena, Italy; 2 Dept. of Medicine, Surgery & Neuroscience, University of Siena, Siena, Italy; 3 Dept. of Neurology, General Hospital of “S. Donato d’Arezzo”, Arezzo, Italy; 4 Unit of Nuclear Medicine, Azienda Ospedaliera Universitaria Senese, Santa Maria alle Scotte General Hospital, Siena, Italy; 5 Unit of Endovascular and Percutaneous Therapy, Clinica Nuestra Senora del Rosario, Madrid, Spain; 6 Dept. Life Sciences, University of Siena, Siena, Italy; 7 Berenson-Allen Center for Non-Invasive Brain Stimulation, Beth Israel Deaconess Medical Center, Harvard Medical School, Boston, Massachusetts, United States of America; University of Düsseldorf, GERMANY

## Abstract

Literature has suggested that changes in brain flow circulation occur in patients with multiple sclerosis. In this study, digital subtraction angiography (DSA) was used to measure the absolute CCT value in MS patients and to correlate its value to age at disease onset and duration, and to expand disability status scale (EDSS). DSA assessment was performed on eighty MS patients and on a control group of forty-four age-matched patients. CCT in MS and control groups was calculated by analyzing the angiographic images. Lesion and brain volumes were calculated in a representative group of MS patients. Statistical correlations among CCT and disease duration, age at disease onset, lesion load, brain volumes and EDSS were considered. A significant difference between CCT in MS patients (mean = 4.9s; sd = 1.27s) and control group (mean = 2.8s; sd = 0.51s) was demonstrated. No significant statistical correlation was found between CCT and the other parameters in all MS patients. Significantly increased CCT value in MS patients suggests the presence of microvascular dysfunctions, which do not depend on clinical and MRI findings. Hemodynamic changes may not be exclusively the result of a late chronic inflammatory process.

## Introduction

Multiple sclerosis (MS) is an inflammatory demyelinating disease of the Central Nervous System (CNS), with presumed autoimmune aetiology, affecting genetically susceptible people. Although different patterns of evolution and variable rates of disability accumulation have been observed, the study of its natural history in a large cohort of patients [1] has suggested that clinical phenotype and disease progression are age dependent. This epidemiological observation raises the possibility that other factors or comorbidities might have an impact on its evolution. A possible disease modifying factor, which varies with both inflammatory process and age, is brain perfusion. A link between MS lesions and abnormalities of small deep white matter veins has long been demonstrated [2] and correlated to the spread of inflammatory mediators across the brain. These mediators would activate local vasoactive factors that cause microvascular damage and brain perfusion alterations. However, the sequence of the pathological events that link the inflammation process to the microvascular changes remain elusive; far from being clarified. [3] Cerebral hypoperfusion is a condition characterized by a decrease in blood supply due to an underlying vascular disorder and usually leads to cerebral hypoxia. Chronic hypoxia might negatively influence disease progression in many different ways [4]. MRI or CT perfusion have proved hypoperfusion both in lesions and in normal-appearing white and grey matter of patients with clinically definite MS. [5–7] Dynamic susceptibility contrast-enhanced (DSC) MRI [8], Arterial Spin Labeling (ASL) technique [9], CT [10–11], radionuclide imaging [12–13] usually provide cerebral perfusion parameters. Contrast-enhanced Ultrasonography is able to estimate CCT as the mean arterio-venous transit time, which is physiologically longer and more variable in comparison to DSA. [14–15] Cerebral blood flow (CBF), cerebral blood volume (CBV) and mean transit time (MTT) are perfusion parameters obtained from the above mentioned techniques and can vary with commercial programs. The results and diagnostic accuracy of all these techniques were validated in comparison to SPECT and DSA. [16] The X-ray imaging equipment can be used to measure hemodynamic function [17] and cerebral circulation time (CCT) [18–22], besides visualizing the morphology [23–24]. DSA and intravenous radionuclide angiography with non-diffusible tracers [17, 25] improved brain scan detection of CCT in normal and pathological conditions. In our study, we obtained velocity estimation from 2D sequence with modern DSA equipment and calculated CCT [20]. The aim of our study is to demonstrate the absolute value of the increased CCT in all MS patients (Progressive form P-MS and Relapsing-Remitting form RR-MS) and the correlation between CCT and Expanded Disability Status Scale (EDSS), CCT and other clinical variables (disease duration, age at disease onset, age, lesion load and brain volumes). To our knowledge, no studies on CCT in multiple sclerosis measured by DSA have yet been published.

## Materials and Methods

Our protocol was approved by the Research Ethics Board of the General Hospital “Santa Maria alle Scotte”, University of Siena, Italy. Written informed consent was obtained from all patients before conducting DSA and venography. From November 2010 to June 2013, eighty patients (mean age 47.48; thirty men and fifty women) with MS underwent DSA before venography in order to evaluate both arterial and venous cerebral vascular aspects. These vascular aspects were qualitative (i.e. morphology), and quantitative (i.e. CCT). Thirty-five MS patients with primary or secondary progressive (P-MS) and forty-five relapsing-remitting (RR-MS) were recruited. Standard demographic and clinical information was acquired for all patients with MS. A detailed medical and familial history of vascular risk factors and venous diseases was obtained from all participants. MS patients filled out a questionnaire about age at disease onset, age at diagnosis, symptoms at disease onset and current and previous therapy. Neurological disability was assessed through EDSS. MS patients with the following criteria were excluded from the study: a relapse or steroid treatment in the 90 days preceding study entry, history of cerebral congenital vascular malformations or cardiovascular diseases, high thrombophilic risk, pregnancy, and contraindications to receive X-ray contrast agent.

As a control group, forty-four age matched patients with sine materia subarachnoid haemorrhage (mean age 50.73; ten men and thirty-four women), who had undergone six to eight (6–8) months DSA follow-up, were retrospectively evaluated in order to confirm the absence of small vessel disease or other pathologic vascular patterns. In particular, these control subjects did not show pathological MRI, clinical and DSA findings (e.g., fibromuscular dysplasia, aneurysms, stenosis, cardio-vascular diseases, arterial-venous malformation, small vessel disease, parenchymal lesions). To assume normality of cardiac output and systemic vascular resistance, all participants (MS and controls) underwent a physical examination, including cardiovascular and respiratory evaluations.

**Fig 1:**
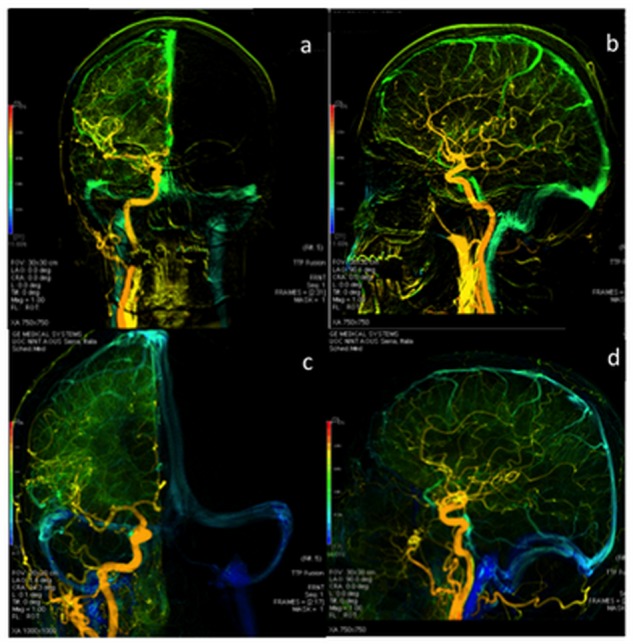
DSA examination: anterior-posterior and lateral views of color-coded right carotid artery of control (a;b) and MS patient (b;c). The blue colour, shown only in MS patients, demonstrates a prolonged CCT. The veins are in green and not blue in the control.

Two blinded observers, an interventional neuroradiologist (SC) who performed the exam (8 years experience) and a neuroradiologist (LM) (22 years experience), performed a detailed interpretation of DSA. Class membership of the subjects was unknown by the observers and their succession was randomly assigned. All DSAs were obtained from anteroposterior, lateral, and working view acquisitions. In case of discrepancies, a third neuroradiologist (EM) (35 years experience) independently reviewed exams in a blinded fashion. A DSA protocol (a 2D DSA acquired at a variable frame rate between 2 and 4 frames/s, with a 4 mL/s contrast injection rate by using power injector) was performed. Intravascular contrast medium injection was done in the internal carotid artery. Images of these cases were analyzed to determine the CCT on the basis of the literature [20]; CCT is defined as the difference in the time between angiographic frames with the maximum concentration in the carotid siphon and in cortical parietal veins on Anterior-Posterior and Lateral views. The same measurement was performed on the right and the left carotid injection and data were compared. Post-processing software (Angioviz; GE Healthcare) was used to colour-code the DSA, according to the time in seconds Fig. 1(a-d).

Cerebral lesion load analysis was carried out on thirty-seven MS patients. This sub-sample of patients was selected on the basis of good quality MRI acquisition. Two-dimensional Fluid Attenuation Inverse Recovery (FLAIR) images were obtained in the axial plain (TE = 104 ms, TR = 9000 ms, slice thickness = 5 mm, slices = 26, matrix = 256×256, voxel size 1×0.9×5, phaseFOV = 87.5). Whole-brain MPRAGE T1 images were obtained in the sagittal plane (TE = 3.61 ms, TR = 2400 ms, flipangle = 8◦, voxel-size = 1.3mm×1.3mm×1.2 mm). A neuroradiologist visually inspected all scans with high expertise in clinical neuroimaging (LM).

### Lesions Detection and Quantification

Segmentation and volume estimation of white matter lesions have been carried out using an in-house pipeline based on Statistical Parametric Mapping software (SPM, Wellcome Department of Cognitive Neurology, Institute of Neurology, University College London; www.fil.ion.ucl.ac.uk/spm/) and ad-hoc scripts developed in the MATLAB scientific computing environment (www.mathworks.com, MathWorks, MA, USA). Differently from previous methods for lesion identification [26–29], our approach allows for lesion identification and evaluation directly in each patient native space, providing better accuracy compared to those procedures implying images spatial normalization to a normative space (e.g. Montreal National Institute—MNI). We proceeded as follows:

1. Coregistration of each participant’s T1 image to his or her respective FLAIR images;
2. Segmentation of T1 images to obtain individual maps for grey/white matter and cerebrospinal fluid (CSF), as well as remove non-brain tissues;
3. Masking of FLAIR images using the white matter maps obtained in Step 2;
4. Spatial normalization of T1 image to the MNI space to obtain individual inverse transformation matrices;
5. Identification of Region-Of-Interest (ROI) to be removed from the FLAIR images in order to decrease the probability of a false positive. Based on previous evidence, we discarded ROIs including the cerebellum (using the Automatic Anatomical Labeling Atlas), brainstem, thalamus, caudate nuclei, putamen (Harvard-Oxford atlas). In order to keep FLAIR images in their native space, these regions, identified in the MNI space, have been transformed to the individual space using the inverse transformation matrices obtained in Step 4;
6. Exclusion of ROIs identified in Step 5 from the <white matter only> FLAIR images created in Step 3;
7. Lesion segmentation by identifying voxels with intensity higher than the 95th percentile of the FLAIR image. Creation of a binary lesion mask for each patient;
8. Visual inspection of lesion localization/extension as identified in the binary mask as compared to the original FLAIR image;
9. Volume quantification in ml using get totals function for SPM.

The process required ~15’ for each patient.

### Total Brain Volume Estimation

In order to verify the existence of a significant correlation between total brain volume and CBF, we also calculated the total brain volume for each participant as well as different volume indexes for grey and white matter, as well as for CBF. The analysis was run in each subject anatomical space using IBASPM toolbox for SPM. Volume was expressed in cm3.

### Statistical Analysis

Reproducibility of the quantitative CCT measurements was examined by means of intraclass correlation coefficients (ICC). The 95% confidence interval of the ICCs (95%CI) was also estimated. Reproducibility analysis was conducted separately for left and right side of ICA within the MS group and the control group. The ICCs were calculated by using two-way mixed model with measures of absolute agreement (ICC3,1), where the subjects were treated as randomly sampled from a larger population and the two neuroradiologists (raters) as fixed factor.

Comparison of the CCT measurements from the right and left internal carotid artery was also performed in MS patients and in control group. A two-way ANOVA test was performed in order to verify if the two raters measured equal values on average of the CCT, if the levels of CCT was equal across the conditions right and left ICA and if the two factors (rater and side) did interact. If the null hypothesis of equal CCT measurements were not rejected, then the series obtained from the right side would have been considered for the study.

Before applying parametric tests (t, ANOVA) the consistence of the hypothesis of normal distribution and of homoscedasticity of the data was verified, by means of the D’Agostino-Pearson omnibus normality test and the Brown-Forsythe’s test, respectively.

If the hypothesis of homoscedasticity was verified, the test statistic under the null hypothesis had Student’s t distribution with n+m-2 degrees of freedom (df), n and m being the sizes of the samples. Instead, if the hypothesis of homoscedasticity was not verified, then the test statistic to compare the two sets of data, under the null hypothesis, had an approximate Student’s t distribution, with a number of df given by the Satterthwaite’s approximation. The 95% confidence interval of the difference in population means was also provided. Two-way ANOVA test was used to study if the factor “gender” and/or its interaction with the factor “group” (MS and controls) can contribute to explaining the variability of the CCT within and between the groups. Within the group of MS patients, the association between CCT, age and the clinical parameters (disease duration, EDSS, age at disease onset) was measured by the Pearson’s correlation coefficient. The corresponding p values of the coefficients were reported. Non-parametric statistics were considered when the normality condition was not met (Mann-Whitney, Spearman correlation coefficient).

The analysis of lesion and brain volume was performed over the sub-sample of thirty-seven MS patients (25RR-MS and 12P-MS). Kolmogorov-Smirnov test and Mann-Whitney test were applied to compare the CCT distributions of this sub-set of patients with the whole MS patients group.

Spearman correlation coefficients were computed to assess the relationship between the volume assessment from the MRI outcomes and CCT measurements.

## Results

**Fig 2:**
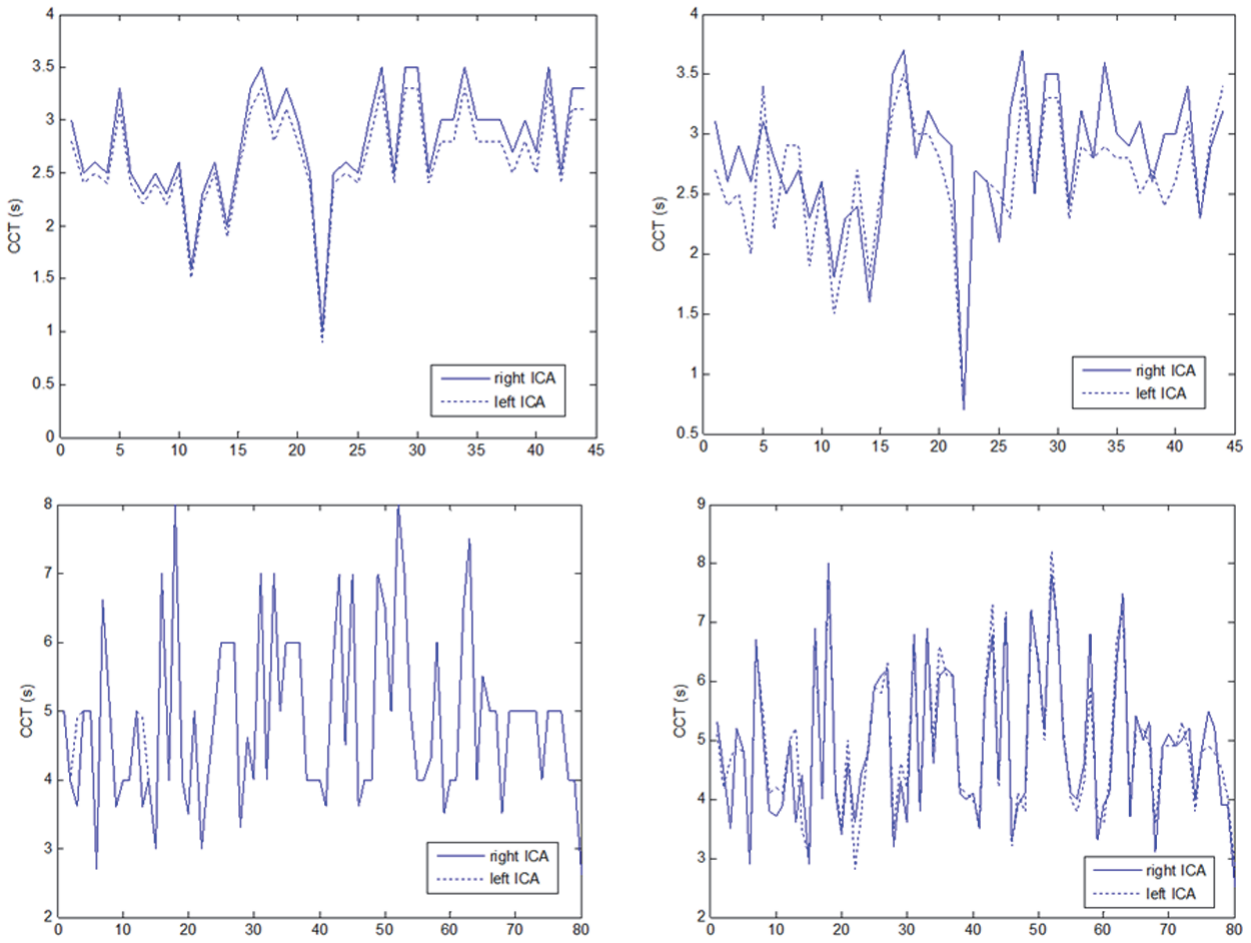
The plots in the panels indicate the close similarity between the CCT evaluations by rater and side. Upper panels: the CCT measurements at the right and left side ICA across the subjects in the control group evaluated by first rater (left panel) and second rater (right panel).

Reproducibility assessments of the CCT measurements by means of the ICC3,1 were calculated for the control group in the right side (ICC3,1 = 0.934; 95%CI = [0.882:0.963]), for the control group in the left side (ICC3,1 = 0.897; 95%CI = [0.819:0.943]), for the MS group in the right side (ICC3,1 = 0.984; 95%CI = [0.974:0.989]) and for the MS group in the left side (ICC3,1 = 0.984; 95%CI = [0.975–0.989]). These findings indicated either strong or almost complete agreement between the two neuroradiologists who performed the CCT evaluations. (Fig. 2)

The first rater’s CCT measures for control group (right side: mean = 2.8, SD = 0.52; left side: mean = 2.6, SD = 0.50) and for MS patients (right side: mean = 4.9, SD = 1.27; left side: mean = 4.9, SD = 1.27) were compared with the corresponding second rater’s CCT measures for control group (right side: mean = 2.8, SD = 0.58; left side: mean = 2.6, SD = 0.54) and for MS group (right side: mean = 4.9, SD = 1.29; left side: mean = 4.9, SD = 1.25).

In the control group a two-way ANOVA with the side ICA (right and left) and rater (the two neuroradiologists) as between subject factors did not reveal main effects over the CCT variability. Neither the side factor (F = 3.71 on 1 and 1 degrees of freedom, p = 0.06), nor the rater factor (F = 0 on 1 and 1 degrees of freedom, p = 0.98) resulted significant. Side effect was not qualified by an interaction between raters and side (F = 0 on 1 and 1 degrees of freedom, p = 0.98). Likewise, for the MS group, neither side effect (F = 0.05 on 1 and 1 degrees of freedom, p = 0.83), nor rater effect (F = 0 on 1 and 1 degrees of freedom, p = 0.96), nor their interaction (F = 0 on 1 and 1 degrees of freedom, p = 0.98) was significant.

This result suggested that neither the laterality nor the rater factors would bias the CCT outcomes.

**Fig 3:**
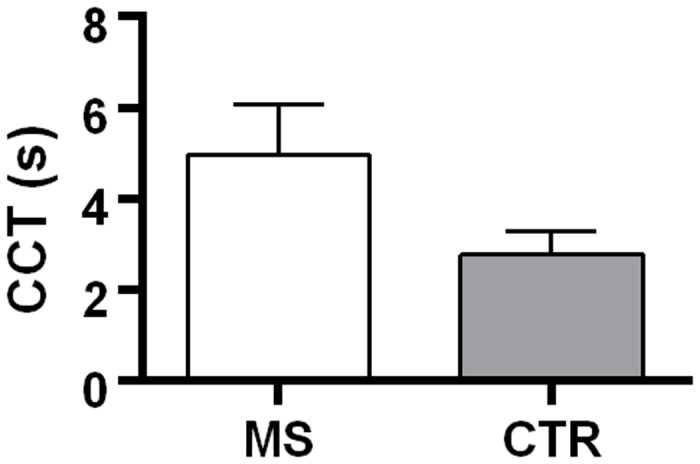
The figure represents the distribution (median and IQR) of the CCT measured in seconds. The control group showed significant lower CCT. The difference between the CCT mean in MS and CTR was 2.09 s with a confidence interval of 95% [1.77 s: 2.41 s].

Statistical analysis was carried out on right side CCT values. There was significant difference between CCT in MS patients (mean = 4.87 s; sd = 1.27s; median = 5 s; IQR = 1.87 s) and control group (mean = 2.78 s; sd = 0.51 s; median = 2.66 s; IQR = 0.72 s) (Fig. 3).

Since the assumption of homoscedasticity was not met, the t test was calculated with a number of degrees of freedom given by Satterthwaite’s approximation (df = 115.22). The t test then resulted: t = 12.88; p < 0.001. The confidence interval 95% of the difference of the CCT means ranged from 1.77s to 2.41s.

The achieved power was 0.83 with medium effect size (0.5) and significant level α = 0.05.

A Mann-Whitney Test was used to compare CCT values between RR-MS (median = 4s; IQR = 1s) and P-MS (median = 5s; IQR = 2s;). No significant difference was observed (U = 658; p = 0.20).

**Fig 4:**
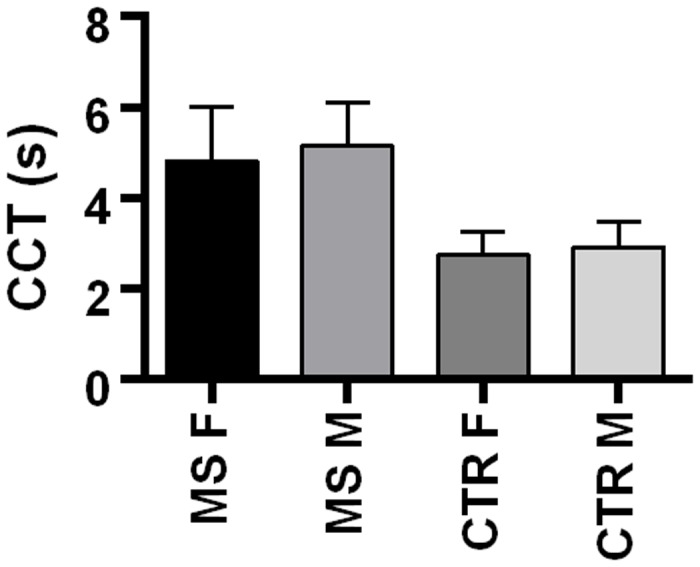
The figure displays the mean and standard deviation of the CCT (in seconds) by gender in MS patients and control group.

No significant statistical difference was revealed between genders in each group (Fig. 4).

The age factor was balanced among groups. Neither the differences in variances, nor the differences in the means were significant, as shown by the Brown-Forsythe’s Test for equality of variances (F = 2.0733, df1 = 2, df2 = 101; p = 0.1311) and by the ANOVA test (F = 2.942, df1 = 2, df2 = 101; p = 0.057), respectively.

The logarithmic transform of the CCT data consented to meet the homoscedasticity condition (F = 2.16; p = 0.09). The two-way ANOVA test with fixed factors “gender” and “group” was then applied to the log-transformed CCT data. Neither the gender factor nor the interaction between gender and group resulted significant to explain the variability of CCT data (p = 0.67 and p = 0.43, respectively). A normal cerebral vascular DSA pattern was observed in subjects of the control group. No stenosis of internal carotid arteries, vertebral arteries, basilar artery or arterial tracts of Willis’ circle were demonstrated by DSA examination in MS patients. The colour-code DSA images obtained as a function of time (s) provided a different vascular map between MS patients and controls. This was a chromatic scale of time based on qualitative assessment. Color-code DSA images revealed a delayed venous phase in MS patients, whilst venous phase was normal in controls. (Fig. 1: a-d).

### CCT & Lesion Volume & Brain Volume

The CCT in the sub-group of thirty-seven patients ranged between 2.6 and 8 s, with median = 5 s, IQR = 1.75 s, mean = 4.8s, SD = 1.32s. A two-sample Kolmogorov-Smirnov test was used to compare the distributions of the values of CCT in this sub-sample and the whole MS group, (K-S = 0.0622; p = 0.99). Moreover, both groups showed same median values (5s) and similar IQR (1.75s vs. 1.87s). Accordingly, Mann-Whitney test demonstrated no significant differences in CCT (U = 1457; p = 0.89). These results indicated that the distribution of CCT did not differ significantly between the two groups of MS patients examined and hence the subset considered for lesion analysis was consistent with the whole MS group.

The average lesion volume was 15.3 ml (SD = 7.41 ml). Mean volume of RR-MS was 15.5 ml (SD = 7.17ml), mean volume of P-MS was 15 ml (SD = 8.20ml).

There was not significant correlation between CCT and lesion volume either in the sample of 37 MS patients (r = 0.0231; p = 0.89), or in the sub-samples of RR-MS (r = 0.0708; p = 0.74) and P-MS (r = -0.1284; p = 0.69) MS forms.

Similarly, the correlation between CCT and brain volume was not significant (r = -0.0802; p = 0.64) in the sub-samples of RR-MS (r = -0.3527; p = 0.08) and P-MS (r = -0.0392; p = 0.90) MS forms.

These results suggested that the variations in the CCT measurements did not interact with the brain and lesion volume assessments.

### CCT & Disease duration EDSS & Disease Onset

The EDSS of the MS patients ranged from 1 to 9.5, the median was 5.25 and IQR = 3.5. Disease duration ranged from 2 to 41 yrs; the median was 13 yrs and IQR 10.5 yrs. The age at disease onset ranged from 14 to 54 yrs; the median was 34 yrs and IQR = 15 yrs (Table 1).

**Table 1:** Clinical Data and CCT values in MS patients and Control Groups.

	MS Patients			
Characteristic	Control Subjects	Whole group	RR-MS	P-MS
Sample size	44	80	45	35
Male-to-Female ratio	10/34	30/50	14/31	16/19
Age yrs.	50.7 (13.12)	47.5 (9.05)	45.3 (9.02)	51.1 (9.18)
EDSS (IQR)*		5.25 (3.5)	3.5 (3)	6 (2.5)
Disease duration yrs.		13.5 (0.96)	11.9 (1.10)	15.1 (1,64)
Age at disease onset yrs.		35.2 (1.97)	32.9 (1.26)	35.2 (1.79)
CCT s	2.8 (0.51)	4.8 (1.27)	4.7 (0.17)	5.1 (0.23)
Therapy:				
No therapy		34	16	18
Natalizumab		10	6	4
Glatiramer acetate		12	8	4
Azathioprine		5	2	3
Interferon β-1a or β-1b		18	12	6
Other therapies		1	1	0

Data are means with standard deviations in parentheses.

* For EDSS median and IQR are instead reported. Other therapy is 4-aminopyridine.

Correlation analysis confirmed the absence of significant association between the variable CCT and other variables. In fact, the corresponding Pearson’s coefficients for the correlations: “CCT vs. Disease duration”, “CCT vs. EDSS”, “CCT vs. age at disease onset”, “CCT vs. Age” were, r = 0.017, r = 0.17, r = -0.12, r = 0.15 respectively, with all the p values largely greater than 0.05 (explicitly, 0.88, 0.12, 0.27, 0.18).

## Discussion

The present study reported a significant CCT increase in MS patients compared to control subjects, indicating a consistent condition of cerebral hypoperfusion. Increase of CCT, and the following cerebral hypoperfusion, vary with blood in-flow reduction due to stenosis and/or parenchymal high resistance, changing PaCO2, or alteration of the cerebral perfusion pressure. Indeed, cerebral perfusion pressure is defined as the difference between systemic arterial pressure and intracranial pressure [17, 30] [13, 16]. Moreover, CCT could vary along with extra-cerebral factors as cardiac output or vascular resistance. In literature, a correlation between narrowing of the artery and delayed cerebral ischemia diagnosed by DSA and CT perfusion has been reported [10, 31]. In addition, Color-coded quantitative DSA has been used to evaluate CCT in patients with steno-occlusive arterial disease before and after endovascular treatment [32].

Many studies have evaluated the absolute quantification of perfusion parameters such as CBV, CBF and MTT, acquired either with MR or CT imaging. The results of these studies revealed that absolute quantification is difficult to measure and often variable [33–34]. Indeed, MTT is an indirect valuable indicator in absolute units (s) of the cerebral circulation since it is the ratio of CBV to CBF (CBV/CBF). MTT measured by dynamic susceptibility contrast-enhanced MRI is calculated by using Fourier transformation (FT) or singular value decomposition deconvolution with correction for effect of tracer delay. It is necessary to point out that MTT is overestimated if FT is used [34]. Another non-invasive technique, such as contrast material-enhanced Ultrasonography, has been used to measure CCT in MS patients. Indeed Mancini et al. have described a prolonged CCT in MS patients by using this technique and considering a carotid artery/jugular vein transit time [14–15]. However, the increased CCT calculated by contrast material-enhanced Ultrasonography could be strongly affected by the pulmonary circulation, even in the presence of normal cardiac output and systemic vascular resistance. On the contrary, CCT measured by DSA is not subordinated by pulmonary circulation since the contrast medium is directly injected in internal carotid artery and the transit between carotid syphon and cortical veins is exclusively taken into account. Thus DSA provides a better representation of the cerebral microcirculation. In the present study, DSA and venography were used to obtain the highest diagnostic accuracy of the hemodynamic alterations and morphology in both arterial and venous vessels. DSA study in MS patients can be performed only in specific research investigations, but it is an invasive technique, and it is not suitable for large-scale clinical studies. Alternatively, non-invasive techniques such as MRI, SPECT, PET, and CT usually provide a large amount of quantitative and morphological data.

In the present study, we have shown that cerebral capillary net studied by DSA was increased in MS patients, suggesting that the increase of CCT could be due to a prolonged capillary phase. Moreover, DSA images have shown a diffuse and homogeneous prolonged capillary and venous phases in both cerebral hemispheres, regardless of the number and site of MS lesions or lesion load and brain volume (Fig. 3). These findings support the hypothesis that the increased CCT depends on microvascular dysfunction and/or neurovascular unit damage. Increased cerebral vessel resistance and/or microvascular dysfunction may lead to chronic hypoxia, which reduces cerebrovascular reserves and subsequent cerebral dysfunctions and higher brain susceptibility to vascular lesions. Microvascular tone, and therefore cerebral perfusion, could be modulated by vasoactive mediators [3, 9].

MRI studies have focused on DWI as a highly sensitive detection tool of acute and chronic tissue changes in MS [35, 36]. Reduced apparent diffusion coefficient (ADC) due to cytotoxic cell swelling has been demonstrated in acute demyelinating lesions [35, 36]. In addition, an alteration in cerebral vasculature has been demonstrated also in normal appearing white matter [6, 7]. Infiltration of vein and capillary walls without adjacent parenchymal inflammation suggests that a form of vasculitis precedes MS lesion development [37–38]. Obliterative vasculitis leads to hypoperfusion and to chronic ischemia, which could alternatively result in diffuse parenchymal dysfunction or damage.

The strong CCT value difference between control group and MS patients explains hypoperfusion, which preempts myelin breakdown and MRI detectable white matter lesions.

The absence of a significant association between CCT and disease duration, disease onset, lesion and brain volume, EDSS and age could suggest that the high intravascular resistance is a constant finding in MS patients, possibly taking place at an early stage of the disease. Therefore, cerebrovascular changes are not solely the result of a late chronic inflammatory process. Indeed, if the microvascular dysfunction was a consequence of lesion load or brain atrophy, high CCT values would be expected to increase with EDSS and disease duration.

In conclusion, the absence of a correlation between lesion volume and CCT confirms that hemodynamic alteration is not related to parenchymal lesion and other MS-linked clinical features, but rather, is a pathognomonic feature of disease.
