# Correction: Cerebral Circulation Time is Prolonged and Not Correlated with EDSS in Multiple Sclerosis Patients: A Study Using Digital Subtracted Angiography

**DOI:** 10.1371/journal.pone.0123731

**Published:** 2015-03-30

**Authors:** The PLOS ONE Staff

Sandra Bracco is not included in the author byline. She should be listed as the ninth author and affiliated with Unit of Neuroimaging and Neurointervention, AOUS, Policlinico “Santa Maria alle Scotte”, Viale Mario Bracci, 16—53100 Siena, Italy. The contributions of this author are noted in the Author Contributions.
